# Diethyl 4-meth­oxyoxalyl-3,5-diphenyl­pyrrolidine-2,2-dicarboxyl­ate

**DOI:** 10.1107/S160053681201392X

**Published:** 2012-04-06

**Authors:** Yan-Qin He, Jin-Quan Chen

**Affiliations:** a Chemical Synthesis and Pollution Control Key Laboratory of Sichuan Province, China West Normal University, Nanchong 637002, People’s Republic of China

## Abstract

In the title compound, C_25_H_27_NO_7_, the pyrrolidine ring exhibits an envelope conformation and the benzene rings form a dihedral angle of 33.47 (11)°. In the crystal, pairs of N—H⋯O hydrogen bonds link the mol­ecules into centrosymmetric dimers. Weak C—H⋯O inter­actions link the dimers into layers parallel to the *bc* plane.

## Related literature

For applications of pyrrolidine derivatives, see: Shih *et al.* (1995 ▶); Enyedy *et al.* (2001 ▶); Kravchenko *et al.* (2005 ▶); Lack *et al.* (2011 ▶).
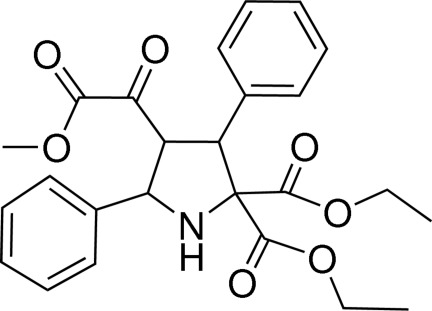


## Experimental

### 

#### Crystal data

C_25_H_27_NO_7_
*M* *_r_* = 453.48
Monoclinic, 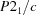

*a* = 8.8779 (3) Å
*b* = 20.3438 (6) Å
*c* = 13.5740 (5) Å
β = 102.213 (3)°
*V* = 2396.12 (14) Å^3^
*Z* = 4
Mo *K*α radiation
μ = 0.09 mm^−1^
*T* = 290 K
0.38 × 0.35 × 0.30 mm

#### Data collection

Oxford Diffraction Gemini S Ultra diffractometer
Absorption correction: multi-scan (*CrysAlis PRO*; Oxford Diffraction, 2010 ▶) *T*_min_ = 0.966, *T*_max_ = 0.973
14990 measured reflections
4887 independent reflections
3129 reflections with *I* > 2σ(*I*)
*R*_int_ = 0.033

#### Refinement

*R*[*F* ^2^ > 2σ(*F* ^2^)] = 0.051
*wR*(*F* ^2^) = 0.133
*S* = 1.02
4887 reflections
305 parameters
8 restraints
H atoms treated by a mixture of independent and constrained refinement
Δρ_max_ = 0.28 e Å^−3^
Δρ_min_ = −0.26 e Å^−3^

### 

Data collection: *CrysAlis PRO* (Oxford Diffraction, 2010 ▶); cell refinement: *CrysAlis PRO*; data reduction: *CrysAlis PRO*; program(s) used to solve structure: *SHELXS97* (Sheldrick, 2008 ▶); program(s) used to refine structure: *SHELXL97* (Sheldrick, 2008 ▶); molecular graphics: *XP* in *SHELXTL* (Sheldrick, 2008 ▶); software used to prepare material for publication: *SHELXL97*.

## Supplementary Material

Crystal structure: contains datablock(s) global, I. DOI: 10.1107/S160053681201392X/cv5272sup1.cif

Structure factors: contains datablock(s) I. DOI: 10.1107/S160053681201392X/cv5272Isup2.hkl

Supplementary material file. DOI: 10.1107/S160053681201392X/cv5272Isup3.cml

Additional supplementary materials:  crystallographic information; 3D view; checkCIF report

## Figures and Tables

**Table 1:** Hydrogen-bond geometry (Å, °)

*D*—H⋯*A*	*D*—H	H⋯*A*	*D*⋯*A*	*D*—H⋯*A*
N1—H1*N*⋯O1^i^	0.88 (2)	2.27 (2)	3.061 (2)	150 (2)
C23—H23⋯O3^ii^	0.93	2.54	3.461 (3)	170
C25—H25⋯O7^iii^	0.93	2.60	3.526 (3)	172

Symmetry codes: (i) 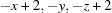; (ii) 
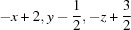
; (iii) 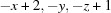.

**Fig. 1.**
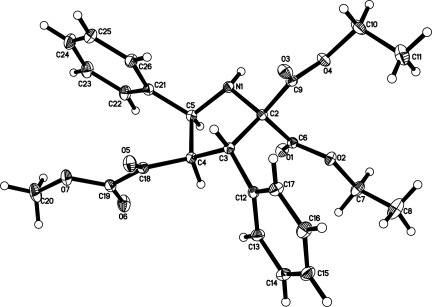
The molecular structure of (I) showing 30% probability displacement ellipsoids and the atomic numbering.

## supplementary crystallographic information

### Comment

Pyrrolidine derivatives have been found to be the core of numerous natural products and pharmaceutical candidates, see: Shih *et al.* (1995); Enyedy *et al.* (2001); Kravchenko *et al.* (2005); Lack *et al.* (2011). Herein, we report the crystal structure of the title compound (I).

The bond lengths and angles in (I) are normal. The pyrrolidine ring exhibits an envelope conformation, with the C2 atom occupying the flap position (Fig. 1). Two benzene rings - C12—C17 and C21—C26, respectively - form a dihedral angle of 33.47 (11)°. In the crystal structure, intermolecular N—H···O hydrogen bonds (Table 1) link the molecules into centrosymmetric dimers, and weak C—H···O interactions (Table 1) link further these dimers into layers parallel to *bc* plane.

### Experimental

To a flame-dried test tube was added *N*,*N*-dimethyl-1-[2-(diphenylphosphino) ferrocenyl]ethylamine (0.04 mmol), copper(II) trifluoromethanesulfonate (0.04 mmol), THF (O.5 ml), benzaldehyde (0.24 mmol) and diethyl *A*-aminomalonate (0.24 mmol) under an argon atmosphere. After the mixture was stirred at room temperature for 2 h, a solution of 2-Oxo-4-phenyl-but-3-enoic acid methyl ester (0.2 mmol) in THF (1.0 ml) was added through a syringe in one portion and the resulting mixture was stirred at room temperature until TLC showed no starting material left. Then, solvent was removed and the residue was purified directly by silica gel column chromatography (eluent: petroleum ether / ethyl acetate = 20/1 to 5/1) to yield product. The colourless single crystals suitable for X-ray diffraction were obtained in methanol solvent by slow evaporation.

### Refinement

N-bound H atoms were located in a difference Fourier map and refined isotropically [N—H = 0.878 (16) Å]. The C-bound H atoms were placed in calculated positions, with C—H = 0.93 - 0.98 Å, and refined using a riding model, with *U*_iso_(H) = 1.2 or 1.5*U*_eq_(C).

### Crystal data

**Table:**

C_25_H_27_NO_7_	*F*(000) = 960
*M**_r_* = 453.48	*D*_x_ = 1.257 Mg m^−^^3^
Monoclinic, *P*2_1_/*c*	Mo *K*α radiation, λ = 0.71073 Å
Hall symbol: -P 2ybc	Cell parameters from 4380 reflections
*a* = 8.8779 (3) Å	θ = 3.2–29.1°
*b* = 20.3438 (6) Å	µ = 0.09 mm^−^^1^
*c* = 13.5740 (5) Å	*T* = 290 K
β = 102.213 (3)°	Block, colorless
*V* = 2396.12 (14) Å^3^	0.38 × 0.35 × 0.30 mm
*Z* = 4	

### Data collection

**Table:**

Oxford Diffraction Gemini S Ultra diffractometer	4887 independent reflections
Radiation source: Enhance (Mo) X-ray Source	3129 reflections with *I* > 2σ(*I*)
Graphite monochromator	*R*_int_ = 0.033
Detector resolution: 15.9149 pixels mm^-1^	θ_max_ = 26.4°, θ_min_ = 3.2°
ω scans	*h* = −11→11
Absorption correction: multi-scan (*CrysAlis PRO*; Oxford Diffraction, 2010)	*k* = −25→25
*T*_min_ = 0.966, *T*_max_ = 0.973	*l* = −16→14
14990 measured reflections	

### Refinement

**Table:**

Refinement on *F*^2^	Primary atom site location: structure-invariant direct methods
Least-squares matrix: full	Secondary atom site location: difference Fourier map
*R*[*F*^2^ > 2σ(*F*^2^)] = 0.051	Hydrogen site location: inferred from neighbouring sites
*wR*(*F*^2^) = 0.133	H atoms treated by a mixture of independent and constrained refinement
*S* = 1.02	*w* = 1/[σ^2^(*F*_o_^2^) + (0.0554*P*)^2^ + 0.3662*P*] where *P* = (*F*_o_^2^ + 2*F*_c_^2^)/3
4887 reflections	(Δ/σ)_max_ < 0.001
305 parameters	Δρ_max_ = 0.28 e Å^−^^3^
8 restraints	Δρ_min_ = −0.26 e Å^−^^3^

### Special details

**Table:**

Geometry. All e.s.d.'s (except the e.s.d. in the dihedral angle between two l.s. planes) are estimated using the full covariance matrix. The cell e.s.d.'s are taken into account individually in the estimation of e.s.d.'s in distances, angles and torsion angles; correlations between e.s.d.'s in cell parameters are only used when they are defined by crystal symmetry. An approximate (isotropic) treatment of cell e.s.d.'s is used for estimating e.s.d.'s involving l.s. planes.
Refinement. Refinement of *F*^2^ against ALL reflections. The weighted *R*-factor *wR* and goodness of fit *S* are based on *F*^2^, conventional *R*-factors *R* are based on *F*, with *F* set to zero for negative *F*^2^. The threshold expression of *F*^2^ > σ(*F*^2^) is used only for calculating *R*-factors(gt) *etc*. and is not relevant to the choice of reflections for refinement. *R*-factors based on *F*^2^ are statistically about twice as large as those based on *F*, and *R*- factors based on ALL data will be even larger.

### Fractional atomic coordinates and isotropic or equivalent isotropic displacement parameters (Å^2^)

**Table:**

	*x*	*y*	*z*	*U*_iso_*/*U*_eq_	
N1	0.9482 (2)	0.04749 (8)	0.86894 (15)	0.0445 (4)	
O1	0.75874 (18)	0.01366 (7)	1.00477 (12)	0.0587 (4)	
O2	0.68505 (17)	0.11814 (7)	1.01209 (11)	0.0537 (4)	
O3	0.90888 (18)	0.20277 (8)	0.85551 (13)	0.0627 (5)	
O4	0.99367 (18)	0.16006 (7)	1.00924 (12)	0.0606 (5)	
O5	0.6663 (2)	0.05129 (8)	0.58463 (13)	0.0646 (5)	
O6	0.5961 (2)	−0.10174 (9)	0.66768 (15)	0.0833 (6)	
O7	0.6680 (2)	−0.07505 (8)	0.52526 (13)	0.0672 (5)	
C2	0.8329 (2)	0.09267 (9)	0.89093 (15)	0.0390 (5)	
C3	0.7135 (2)	0.09231 (9)	0.78941 (15)	0.0393 (5)	
H3	0.7590	0.1168	0.7408	0.047*	
C4	0.7112 (2)	0.01965 (9)	0.75916 (16)	0.0416 (5)	
H4	0.6324	−0.0027	0.7877	0.050*	
C5	0.8726 (2)	−0.00897 (10)	0.81100 (16)	0.0427 (5)	
H5	0.8575	−0.0436	0.8582	0.051*	
C6	0.7574 (2)	0.06980 (10)	0.97687 (16)	0.0418 (5)	
C7	0.5949 (3)	0.10022 (14)	1.0861 (2)	0.0740 (8)	
H7A	0.5254	0.0644	1.0608	0.089*	
H7B	0.6625	0.0862	1.1483	0.089*	
C8	0.5078 (5)	0.15783 (19)	1.1041 (3)	0.1267 (13)	
H8A	0.4510	0.1746	1.0409	0.190*	
H8B	0.5773	0.1910	1.1372	0.190*	
H8C	0.4374	0.1460	1.1460	0.190*	
C9	0.9123 (2)	0.15908 (11)	0.91495 (18)	0.0453 (5)	
C10	1.0665 (3)	0.22220 (14)	1.0448 (2)	0.0809 (9)	
H10A	1.1018	0.2439	0.9902	0.097*	
H10B	1.1555	0.2140	1.0986	0.097*	
C11	0.9557 (4)	0.26554 (15)	1.0823 (3)	0.1000 (11)	
H11A	0.8684	0.2741	1.0287	0.150*	
H11B	1.0053	0.3063	1.1056	0.150*	
H11C	0.9219	0.2442	1.1369	0.150*	
C12	0.5595 (2)	0.12441 (10)	0.79130 (15)	0.0412 (5)	
C13	0.4339 (2)	0.09043 (12)	0.81017 (19)	0.0570 (6)	
H13	0.4396	0.0450	0.8180	0.068*	
C14	0.3000 (3)	0.12289 (14)	0.8176 (2)	0.0704 (7)	
H14	0.2169	0.0991	0.8307	0.084*	
C15	0.2887 (3)	0.18935 (14)	0.8060 (2)	0.0683 (7)	
H15	0.1988	0.2110	0.8118	0.082*	
C16	0.4104 (3)	0.22371 (13)	0.7859 (2)	0.0671 (7)	
H16	0.4033	0.2691	0.7776	0.080*	
C17	0.5440 (3)	0.19164 (10)	0.77789 (18)	0.0545 (6)	
H17	0.6255	0.2157	0.7631	0.065*	
C18	0.6739 (2)	0.00889 (10)	0.64679 (17)	0.0459 (5)	
C19	0.6410 (3)	−0.06290 (12)	0.61527 (19)	0.0533 (6)	
C20	0.6394 (4)	−0.14192 (14)	0.4894 (2)	0.0933 (10)	
H20A	0.6611	−0.1457	0.4232	0.140*	
H20B	0.5335	−0.1530	0.4866	0.140*	
H20C	0.7048	−0.1714	0.5345	0.140*	
C21	0.9705 (2)	−0.03601 (10)	0.74167 (17)	0.0466 (5)	
C22	0.9944 (3)	−0.10277 (12)	0.7365 (2)	0.0651 (7)	
H22	0.9494	−0.1311	0.7757	0.078*	
C23	1.0847 (4)	−0.12831 (15)	0.6737 (2)	0.0820 (9)	
H23	1.0998	−0.1734	0.6709	0.098*	
C24	1.1512 (4)	−0.08695 (18)	0.6163 (2)	0.0861 (9)	
H24	1.2127	−0.1040	0.5748	0.103*	
C25	1.1283 (3)	−0.02117 (16)	0.6190 (2)	0.0783 (8)	
H25	1.1730	0.0067	0.5790	0.094*	
C26	1.0380 (3)	0.00470 (12)	0.68185 (19)	0.0611 (6)	
H26	1.0228	0.0499	0.6836	0.073*	
H1N	1.016 (2)	0.0357 (10)	0.9232 (14)	0.054 (7)*	

### Atomic displacement parameters (Å^2^)

**Table:**

	*U*^11^	*U*^22^	*U*^33^	*U*^12^	*U*^13^	*U*^23^
N1	0.0426 (10)	0.0438 (10)	0.0464 (12)	0.0056 (8)	0.0077 (9)	−0.0022 (9)
O1	0.0719 (11)	0.0459 (9)	0.0627 (11)	0.0056 (7)	0.0240 (8)	0.0149 (8)
O2	0.0648 (10)	0.0496 (9)	0.0533 (10)	0.0054 (7)	0.0277 (8)	0.0016 (7)
O3	0.0745 (11)	0.0482 (9)	0.0640 (11)	−0.0176 (8)	0.0113 (9)	0.0053 (9)
O4	0.0645 (10)	0.0603 (10)	0.0520 (10)	−0.0165 (8)	0.0009 (8)	−0.0057 (8)
O5	0.0861 (12)	0.0569 (10)	0.0495 (10)	0.0043 (8)	0.0110 (9)	0.0064 (8)
O6	0.1233 (17)	0.0567 (11)	0.0763 (14)	−0.0250 (10)	0.0357 (12)	−0.0118 (10)
O7	0.0863 (12)	0.0633 (11)	0.0497 (11)	0.0081 (9)	0.0093 (9)	−0.0136 (9)
C2	0.0406 (11)	0.0356 (10)	0.0417 (12)	−0.0003 (8)	0.0104 (9)	0.0006 (9)
C3	0.0429 (11)	0.0374 (11)	0.0384 (12)	−0.0001 (8)	0.0106 (9)	0.0025 (9)
C4	0.0456 (12)	0.0371 (11)	0.0438 (13)	−0.0015 (8)	0.0131 (9)	0.0003 (9)
C5	0.0505 (12)	0.0372 (11)	0.0418 (12)	0.0025 (9)	0.0131 (10)	0.0036 (9)
C6	0.0425 (11)	0.0418 (12)	0.0402 (12)	0.0015 (9)	0.0066 (9)	0.0012 (10)
C7	0.0804 (18)	0.0894 (19)	0.0637 (18)	0.0056 (14)	0.0409 (15)	0.0081 (15)
C8	0.142 (2)	0.132 (2)	0.129 (2)	0.0343 (16)	0.0808 (17)	−0.0039 (16)
C9	0.0423 (12)	0.0439 (12)	0.0519 (15)	−0.0020 (9)	0.0152 (10)	−0.0022 (11)
C10	0.087 (2)	0.0787 (19)	0.072 (2)	−0.0357 (16)	0.0045 (16)	−0.0169 (16)
C11	0.141 (3)	0.072 (2)	0.085 (2)	−0.012 (2)	0.019 (2)	−0.0180 (18)
C12	0.0449 (12)	0.0424 (11)	0.0368 (12)	0.0007 (9)	0.0094 (9)	0.0013 (9)
C13	0.0479 (13)	0.0519 (13)	0.0731 (18)	−0.0032 (10)	0.0171 (12)	−0.0031 (12)
C14	0.0472 (14)	0.0823 (19)	0.085 (2)	−0.0060 (13)	0.0218 (13)	−0.0096 (16)
C15	0.0543 (15)	0.085 (2)	0.0671 (18)	0.0218 (13)	0.0154 (13)	−0.0022 (15)
C16	0.0718 (17)	0.0548 (15)	0.0766 (19)	0.0199 (13)	0.0201 (14)	0.0073 (13)
C17	0.0546 (14)	0.0469 (13)	0.0642 (16)	0.0049 (10)	0.0171 (12)	0.0077 (11)
C18	0.0439 (12)	0.0464 (12)	0.0471 (13)	0.0020 (9)	0.0087 (10)	−0.0010 (11)
C19	0.0553 (14)	0.0530 (14)	0.0491 (15)	0.0012 (11)	0.0056 (11)	−0.0052 (12)
C20	0.131 (3)	0.0688 (18)	0.072 (2)	0.0200 (17)	0.0035 (19)	−0.0275 (16)
C21	0.0476 (12)	0.0470 (13)	0.0442 (13)	0.0067 (9)	0.0072 (10)	−0.0009 (10)
C22	0.0812 (18)	0.0505 (14)	0.0649 (17)	0.0158 (12)	0.0183 (14)	−0.0015 (12)
C23	0.102 (2)	0.0648 (18)	0.080 (2)	0.0265 (16)	0.0218 (18)	−0.0160 (16)
C24	0.083 (2)	0.103 (2)	0.077 (2)	0.0192 (18)	0.0290 (17)	−0.025 (2)
C25	0.0768 (19)	0.100 (2)	0.0669 (19)	−0.0079 (16)	0.0358 (15)	−0.0105 (17)
C26	0.0670 (16)	0.0576 (15)	0.0631 (17)	−0.0018 (12)	0.0236 (13)	−0.0036 (13)

### Geometric parameters (Å, º)

**Table:**

N1—C2	1.453 (2)	C10—H10A	0.9700
N1—C5	1.472 (3)	C10—H10B	0.9700
N1—H1N	0.878 (16)	C11—H11A	0.9600
O1—C6	1.203 (2)	C11—H11B	0.9600
O2—C6	1.318 (2)	C11—H11C	0.9600
O2—C7	1.457 (3)	C12—C13	1.381 (3)
O3—C9	1.196 (3)	C12—C17	1.383 (3)
O4—C9	1.330 (3)	C13—C14	1.382 (3)
O4—C10	1.455 (3)	C13—H13	0.9300
O5—C18	1.198 (3)	C14—C15	1.362 (4)
O6—C19	1.187 (3)	C14—H14	0.9300
O7—C19	1.317 (3)	C15—C16	1.362 (4)
O7—C20	1.449 (3)	C15—H15	0.9300
C2—C9	1.527 (3)	C16—C17	1.378 (3)
C2—C6	1.536 (3)	C16—H16	0.9300
C2—C3	1.551 (3)	C17—H17	0.9300
C3—C12	1.520 (3)	C18—C19	1.533 (3)
C3—C4	1.533 (3)	C20—H20A	0.9600
C3—H3	0.9800	C20—H20B	0.9600
C4—C18	1.507 (3)	C20—H20C	0.9600
C4—C5	1.569 (3)	C21—C22	1.379 (3)
C4—H4	0.9800	C21—C26	1.382 (3)
C5—C21	1.513 (3)	C22—C23	1.388 (4)
C5—H5	0.9800	C22—H22	0.9300
C7—C8	1.453 (4)	C23—C24	1.364 (4)
C7—H7A	0.9700	C23—H23	0.9300
C7—H7B	0.9700	C24—C25	1.355 (4)
C8—H8A	0.9600	C24—H24	0.9300
C8—H8B	0.9600	C25—C26	1.392 (4)
C8—H8C	0.9600	C25—H25	0.9300
C10—C11	1.489 (4)	C26—H26	0.9300
			
C2—N1—C5	110.00 (16)	C10—C11—H11A	109.5
C2—N1—H1N	112.7 (15)	C10—C11—H11B	109.5
C5—N1—H1N	112.8 (14)	H11A—C11—H11B	109.5
C6—O2—C7	116.37 (18)	C10—C11—H11C	109.5
C9—O4—C10	116.42 (19)	H11A—C11—H11C	109.5
C19—O7—C20	115.8 (2)	H11B—C11—H11C	109.5
N1—C2—C9	106.88 (16)	C13—C12—C17	117.3 (2)
N1—C2—C6	113.85 (16)	C13—C12—C3	123.52 (18)
C9—C2—C6	111.21 (17)	C17—C12—C3	119.14 (18)
N1—C2—C3	101.17 (16)	C12—C13—C14	121.0 (2)
C9—C2—C3	113.06 (16)	C12—C13—H13	119.5
C6—C2—C3	110.31 (15)	C14—C13—H13	119.5
C12—C3—C4	117.24 (16)	C15—C14—C13	120.6 (2)
C12—C3—C2	115.56 (17)	C15—C14—H14	119.7
C4—C3—C2	102.13 (15)	C13—C14—H14	119.7
C12—C3—H3	107.1	C16—C15—C14	119.4 (2)
C4—C3—H3	107.1	C16—C15—H15	120.3
C2—C3—H3	107.1	C14—C15—H15	120.3
C18—C4—C3	113.50 (17)	C15—C16—C17	120.4 (2)
C18—C4—C5	112.60 (17)	C15—C16—H16	119.8
C3—C4—C5	106.19 (15)	C17—C16—H16	119.8
C18—C4—H4	108.1	C16—C17—C12	121.4 (2)
C3—C4—H4	108.1	C16—C17—H17	119.3
C5—C4—H4	108.1	C12—C17—H17	119.3
N1—C5—C21	111.04 (17)	O5—C18—C4	125.16 (19)
N1—C5—C4	102.72 (15)	O5—C18—C19	120.7 (2)
C21—C5—C4	116.51 (17)	C4—C18—C19	114.14 (19)
N1—C5—H5	108.8	O6—C19—O7	125.5 (2)
C21—C5—H5	108.8	O6—C19—C18	122.4 (2)
C4—C5—H5	108.8	O7—C19—C18	112.1 (2)
O1—C6—O2	124.6 (2)	O7—C20—H20A	109.5
O1—C6—C2	123.36 (19)	O7—C20—H20B	109.5
O2—C6—C2	111.96 (17)	H20A—C20—H20B	109.5
C8—C7—O2	107.7 (2)	O7—C20—H20C	109.5
C8—C7—H7A	110.2	H20A—C20—H20C	109.5
O2—C7—H7A	110.2	H20B—C20—H20C	109.5
C8—C7—H7B	110.2	C22—C21—C26	118.0 (2)
O2—C7—H7B	110.2	C22—C21—C5	120.3 (2)
H7A—C7—H7B	108.5	C26—C21—C5	121.66 (19)
C7—C8—H8A	109.5	C21—C22—C23	121.0 (3)
C7—C8—H8B	109.5	C21—C22—H22	119.5
H8A—C8—H8B	109.5	C23—C22—H22	119.5
C7—C8—H8C	109.5	C24—C23—C22	119.7 (3)
H8A—C8—H8C	109.5	C24—C23—H23	120.1
H8B—C8—H8C	109.5	C22—C23—H23	120.1
O3—C9—O4	124.67 (19)	C25—C24—C23	120.5 (3)
O3—C9—C2	124.7 (2)	C25—C24—H24	119.7
O4—C9—C2	110.54 (18)	C23—C24—H24	119.7
O4—C10—C11	110.4 (2)	C24—C25—C26	120.0 (3)
O4—C10—H10A	109.6	C24—C25—H25	120.0
C11—C10—H10A	109.6	C26—C25—H25	120.0
O4—C10—H10B	109.6	C21—C26—C25	120.7 (2)
C11—C10—H10B	109.6	C21—C26—H26	119.6
H10A—C10—H10B	108.1	C25—C26—H26	119.6
			
C5—N1—C2—C9	−159.71 (17)	C9—O4—C10—C11	84.5 (3)
C5—N1—C2—C6	77.1 (2)	C4—C3—C12—C13	28.5 (3)
C5—N1—C2—C3	−41.2 (2)	C2—C3—C12—C13	−92.0 (2)
N1—C2—C3—C12	168.35 (16)	C4—C3—C12—C17	−154.38 (19)
C9—C2—C3—C12	−77.7 (2)	C2—C3—C12—C17	85.1 (2)
C6—C2—C3—C12	47.5 (2)	C17—C12—C13—C14	−1.5 (4)
N1—C2—C3—C4	39.90 (18)	C3—C12—C13—C14	175.6 (2)
C9—C2—C3—C4	153.85 (16)	C12—C13—C14—C15	0.3 (4)
C6—C2—C3—C4	−80.93 (19)	C13—C14—C15—C16	0.6 (4)
C12—C3—C4—C18	82.3 (2)	C14—C15—C16—C17	−0.3 (4)
C2—C3—C4—C18	−150.29 (17)	C15—C16—C17—C12	−1.0 (4)
C12—C3—C4—C5	−153.43 (17)	C13—C12—C17—C16	1.9 (4)
C2—C3—C4—C5	−26.1 (2)	C3—C12—C17—C16	−175.4 (2)
C2—N1—C5—C21	149.70 (17)	C3—C4—C18—O5	9.5 (3)
C2—N1—C5—C4	24.5 (2)	C5—C4—C18—O5	−111.1 (2)
C18—C4—C5—N1	127.43 (18)	C3—C4—C18—C19	−169.00 (17)
C3—C4—C5—N1	2.6 (2)	C5—C4—C18—C19	70.3 (2)
C18—C4—C5—C21	5.8 (2)	C20—O7—C19—O6	−0.1 (4)
C3—C4—C5—C21	−118.94 (19)	C20—O7—C19—C18	−179.9 (2)
C7—O2—C6—O1	−3.3 (3)	O5—C18—C19—O6	−153.7 (2)
C7—O2—C6—C2	173.36 (18)	C4—C18—C19—O6	24.9 (3)
N1—C2—C6—O1	−19.7 (3)	O5—C18—C19—O7	26.1 (3)
C9—C2—C6—O1	−140.5 (2)	C4—C18—C19—O7	−155.34 (19)
C3—C2—C6—O1	93.2 (2)	N1—C5—C21—C22	133.7 (2)
N1—C2—C6—O2	163.56 (16)	C4—C5—C21—C22	−109.2 (2)
C9—C2—C6—O2	42.8 (2)	N1—C5—C21—C26	−46.4 (3)
C3—C2—C6—O2	−83.5 (2)	C4—C5—C21—C26	70.7 (3)
C6—O2—C7—C8	−171.9 (3)	C26—C21—C22—C23	0.5 (4)
C10—O4—C9—O3	8.3 (3)	C5—C21—C22—C23	−179.6 (2)
C10—O4—C9—C2	−175.66 (19)	C21—C22—C23—C24	0.1 (4)
N1—C2—C9—O3	96.2 (2)	C22—C23—C24—C25	−0.8 (5)
C6—C2—C9—O3	−139.0 (2)	C23—C24—C25—C26	0.8 (5)
C3—C2—C9—O3	−14.3 (3)	C22—C21—C26—C25	−0.5 (4)
N1—C2—C9—O4	−79.9 (2)	C5—C21—C26—C25	179.6 (2)
C6—C2—C9—O4	45.0 (2)	C24—C25—C26—C21	−0.1 (4)
C3—C2—C9—O4	169.70 (17)		

### Hydrogen-bond geometry (Å, º)

**Table:**

*D*—H···*A*	*D*—H	H···*A*	*D*···*A*	*D*—H···*A*
N1—H1*N*···O1^i^	0.88 (2)	2.27 (2)	3.061 (2)	150 (2)
C23—H23···O3^ii^	0.93	2.54	3.461 (3)	170
C25—H25···O7^iii^	0.93	2.60	3.526 (3)	172

Symmetry codes: (i) −*x*+2, −*y*, −*z*+2; (ii) −*x*+2, *y*−1/2, −*z*+3/2; (iii) −*x*+2, −*y*, −*z*+1.
